# Incidence, Severity and Impact of Influenza: a joint meeting organised by the ISIRV Epidemiology Group and ECDC, Stockholm, 2019

**DOI:** 10.2807/1560-7917.ES.2019.24.23.1900348

**Published:** 2019-06-06

**Authors:** Barbara Rath, Pasi Penttinen

**Affiliations:** 1 International Society for Influenza and Other Respiratory Viruses (ISIRV) Epidemiology Group, United Kingdom; 2 Vienna Vaccine Safety Initiative, Berlin, Germany; 3 European Centre for Disease Prevention and Control (ECDC), Stockholm, Sweden

**Keywords:** influenza, burden, severity, impact, global health, epidemiology

The International Society for Influenza and Other Respiratory Viruses (ISIRV) Epidemiology Group and the European Centre for Disease Prevention and Control (ECDC) held a joint meeting at the ECDC facilities in Stockholm on 16–18 January 2019. This meeting followed the ‘Incidence, Severity and Impact of Influenza’ meeting in Paris and the ISIRV Epidemiology Group Panel at Options XI in Chicago in 2016.

The 2019 meeting focused on defining, assessing and monitoring the incidence, severity and impact of influenza. The organisers aimed at building bridges between the epidemiology, modelling, public health and clinical communities. The meeting was organised around six core topics: (i) linking clinical research and surveillance, (ii) what happens outside the hospital, (iii) learning from ‘real-world’ clinical and patient data (iv) predicting outcomes on the individual and population level (v) advancing public health surveillance of severity and (vi) surveillance of severity in low resource or crowded settings.

## Initial keynote lectures

Mike Catchpole, Chief Scientist of ECDC, set the stage with a comprehensive overview highlighting the need for enhanced surveillance and risk assessment in the context of global communicable disease threats. Based on extensive review by the International Health Regulations (IHR) Committee following the 2009 influenza A(H1N1) pandemic, the World Health Organization (WHO) was tasked with developing a consistent and interoperable influenza severity assessment terminology and methodology. Katelijn Vandemaele (WHO Geneva, Switzerland) emphasised the need to capture both individual-level clinical severity, as well as the impact on a population overall. International comparisons of severity are often based on influenza-attributable hospitalisations and mortality. These assessments face a number of challenges, such as different criteria for hospitalisation and/or laboratory confirmation, and surveillance infrastructure, as presented by Danielle Iuliano (Centers for Disease Control and Prevention (CDC), Atlanta, United States (US)).

## Linking clinical research and surveillance

The first session aimed at bridging the gap between the outcomes measured in clinical trials and those detected in public-health surveillance systems. After the 2009 pandemic, several large-scale consortia were launched to improve preparedness for epidemics and outbreak scenarios, focused on standardised emergency protocols (e.g. International Severe Acute Respiratory and Emerging Infection Consortium (ISARIC)), clinical trial infrastructure (e.g. Platform for European Preparedness Against (Re-)emerging Epidemics (PREPARE) in Europe, Australian Partnership for Preparedness Research on Infectious Diseases Emergencies (APPRISE) in Australia) and mechanisms to fund urgent research priorities (e.g. Global Research Collaboration for Infectious Disease Preparedness (GloPid-R)). Gail Carson (Oxford University/ISARIC, Oxford, United Kingdom (UK)) provided an overview of existing networks, highlighting important areas that still need to be addressed: the variety of case definitions currently in use, the lack of coordination between clinical teams, the inconsistent use of International Statistical Classification of Diseases and Related Health Problems (ICD) codes (including with regards to causes of death) and the question of what feasible data can be collected and will translate into quality improvement and the timely monitoring of influenza activity.

Siri Helene Hauge (Norwegian Institute of Public Health, Oslo, Norway) presented a register-based study encompassing all general practitioners (GPs) and emergency rooms in Norway, showing a substantial number of visits from healthy young adults. Ben Cowling (Hong Kong University/ISIRV, Hong Kong Special Administrative Region, People’s Republic of China) described epidemiological methods linking weekly burden, severity and impact of influenza that facilitate the prediction of excess mortality during ongoing influenza seasons (referred to as ‘now-casting’), suggesting that future models may need to account for population immunity. This topic was further explored by Deepali Kumar (University of Toronto, Toronto, Canada), who studies the impact of influenza in immunocompromised individuals and presented a recent prospective multi-centre study in transplant recipients aimed at identifying markers of severity. The factors that were positively associated with severe outcomes included advanced age, influenza A infection and hospital-acquisition of influenza. Early-onset (< 48 hours) antiviral therapy and influenza immunisation were both found to be protective, and viral shedding was significantly prolonged, especially in unvaccinated transplant recipients and those requiring intensive care [1].

## What happens outside the hospital?

Tim Uyeki (CDC, Atlanta, US) emphasised that host factors, virology, access to care, vaccination and antiviral treatment all influence clinical severity. An important proportion of individual-level disease burden, including mortality, may go undetected in hospital-based surveillance and needs to be characterised further. Cases of asymptomatic influenza and atypical presentations (such as stroke and myocardial infarction) may never be identified as influenza-related [2]. The issue of under-recognised influenza mortality was investigated in-depth by Jesús Castilla (Institute of Public Health of Navarra, Spain), who presented recent data from post-mortem screenings for influenza in funeral parlours in Spain. His group, which is part of the Integrated Monitoring of Vaccines in Europe (I-Move) project, studies the hidden mortality associated with influenza infection in ageing populations. Andrew Hayward, (University College London, UK) agreed that reported cases of influenza may only represent the tip of the iceberg. Indicators of severity should include a/oligosymptomatic patients and patients who do not seek medical care. The Flu Watch Study of households linked to the UK GP network confirmed that influenza infection may accelerate mortality in patients with underlying conditions [3]. Edgar Mojica (National Autonomous University of Mexico, Mexico City, Mexico) presented innovative bioinformatics methods (‘space-time cubes’) to detect geographical trends of influenza activity outside the hospital. Using temporo-spatial datasets available in Mexico, he was able to identify regional patterns that may help stakeholders to monitor influenza activity over time.

## Learning from ‘real-world’ clinical and patient data

One of the key aspects in the collection of individual patient data is the specification of relevant clinical endpoints, which ideally should be compatible with endpoints measured in clinical trials and observational studies. Regulatory and public health agencies are increasingly interested in ‘real-world’ evidence of disease dynamics and the effect of antivirals and vaccines on the individual. The US Food and Drug Administration (FDA) takes the position that interventions should elicit a ‘positive, clinically meaningful effect on how an individual feels, functions or survives.’ Elektra Papadopoulos (FDA, Silver Spring, US) provided insight into criteria and pathways for the development of clinically relevant outcome measures from a regulatory viewpoint, including for patient reported outcomes. The session continued with a presentation by Barbara Rath (Vienna Vaccine Safety Initiative (ViVI)/ ISIRV, Berlin, Germany) on the integration of a mobile health (m-health) tool, the ViVI Disease Severity Score, to determine clinical outcomes in routine clinical settings. The Partnering for Enhanced Digital Surveillance of Influenza-like Disease and the Effect of Antivirals and Vaccines (PEDSIDEA) project offers the opportunity to gather real-time information on individual-level disease severity across different settings and age groups, including children [4]. Daniela Paolotti (Institute for Scientific Interchange Foundation, Turin, Italy) explored the use of voluntary self-reporting as a means to monitor influenza disease activity in populations, linking data from various participatory networks (e.g. Influenzanet). She discussed the challenges of self-reporting, which may be impacted by numerous factors, including awareness of symptoms in self and others, variability of access to and time for reporting, individual interpretations and lack of objective comparators. In the absence of unbiased laboratory confirmation, self-reported symptoms may be triggered by other pathogens or confounded by latent symptoms and underlying conditions. George Milne (University of Western Australia, Perth, Australia) presented epidemiological methods used in Australia to account for population mobility, household/school/work and transmission. He was able to show that high-dose and adjuvanted vaccines are effective in reducing health outcomes in older adults and that vaccination with these vaccines is cost-effective in comparison to a quadrivalent influenza vaccine [5].

## Predicting outcomes on the individual and population level

Predicting individual-level outcomes of influenza infections remains a challenge. Yuelong Shu (Sun Yat-sen University, Guangzhou, People’s Republic of China) reviewed the current status of research on biomarkers (including host and virulence factors) that may predict individual-level severity in influenza patients. For avian and pandemic influenza viruses, a number of genetic markers have been proposed to predict disease severity in mammals and/or humans [6].

Predicting the population-level impact of influenza seasonal activity has long been considered impossible. Since the 2013–14 influenza season, the US CDC has been working to improve mathematical forecasting models (e.g. FluSight). Matthew Biggerstaff (CDC, Atlanta, US) showed that short-term forecasting has since become feasible [7]. Real-time open-data sourcing may further improve forecasting accuracy, and the utility of compiling various models into ‘ensemble’ models will be the logical next step. Gideon Emokuele (CDC, Nairobi, Kenya) provided insight into influenza disease burden in the context of competing priorities in Sub-Saharan Africa. Due to lack of documentation of causes of death, cause-specific mortality estimates are largely based on verbal autopsies, and need to be further refined to understand the true human and economic burden. Most countries still do not have routine access to influenza testing and thus rely on limited data from influenza surveillance sites. Despite these challenges, eight countries in Sub-Saharan Africa have been able to produce estimates for influenza-associated hospitalisations and two countries for mortality. In the WHO Eastern Mediterranean Region (WHO-EMRO), comprising 22 mostly Northern African and Middle Eastern countries, 19 countries have established a surveillance system for influenza-like illness (ILI) or severe acute respiratory syndrome (SARI) and work is ongoing to implement the Pandemic Influenza Severity Assessment (PISA) methodology in ten of these countries. Mohamed Elhakim (WHO-EMRO, Cairo, Egypt) described how several countries in the Region are currently engulfed in important crises, hampering the effective improvement of surveillance systems. Monthly regional bulletins are published on the WHO website.

## Advancing public health surveillance of severity

ECDC complements the sentinel surveillance of EU Member States with surveillance of intensive-care-unit and hospital-admitted influenza-confirmed cases, monitoring all-cause deaths from vital statistics in EU countries and monitoring vaccine effectiveness during influenza seasons. Pasi Penttinen (ECDC, Stockholm, Sweden) suggested that improving coverage and completeness of reporting remains an ongoing challenge, and preparedness for special studies during the first phase of a pandemic should be enhanced. Jens Nielsen (Statens Serum Institut, Copenhagen, Denmark) described how the European Monitoring of Excess Mortality for Public Health Action (EuroMoMo) network collects data on weekly all-cause deaths from 24 participating European countries. The excess and influenza-attributable mortality during the 2017/18 season, with mainly influenza B circulation, was comparable to A(H3N2) seasons. Carrie Reed (CDC, Atlanta, US) described how her institution is supporting improvements in surveillance at the state level, including burden and vaccination-averted burden estimates. She indicated that severity assessments are further improved by stratification by age, state/region and type/ subtype, and noted that publicly available disease burden and vaccination-averted disease burden estimates have been useful in communicating the public health impact of influenza. She also stated that cross-organisation and interdisciplinary collaboration are necessary for (near) real-time estimates.

Cheryl Jones (University of Melbourne, Melbourne, Australia) turned attention to specific risk groups and non-respiratory symptoms that may be missed during routine surveillance. In Australia, clinical networks have been helpful in determining the incidence of encephalitis/encephalopathy and other forms of influenza-associated neurological disease in children (e.g. Paediatric Active Enhanced Disease Surveillance (PAEDS)/Adverse Childhood Experiences (ACE) study). She noted that severe central nervous system complications in infants and younger children were associated with high morbidity and mortality, though they were often undetected by influenza surveillance systems [8].

## Surveillance of severity in low-resource or crowded settings

Assessing the impact of influenza on low-middle-income countries (LMIC) remains particularly challenging. Lack of seasonality in some countries renders time-series modelling unfeasible. Cheryl Cohen (National Institute for Communicable Diseases, Johannesburg, South Africa) presented an overview of her experience studying the impact of influenza in underserved and high-risk populations. This includes specific risk factors for severe influenza that are less prevalent in high-income settings such as HIV, tuberculosis and malnutrition. Jean-Michel Heraud (Institut Pasteur de Madagascar, Antananarivo, Madagascar), agreed that surveillance and disease burden assessments can be done in low-resource environments; however, more cost-effectiveness studies are warranted to further improve the prevention and control of influenza infections in underserved communities [9]. Ziad Memish (Prince Mohammed bin Abdulaziz Hospital, Riyadh, Kingdom of Saudi Arabia) followed with an overview of acute respiratory infection epidemiology and measures taken for disease control during pilgrimages and mass gatherings in Saudi Arabia. He recommended Post-Hajj cohort studies to further evaluate the impact of the Hajj on the acquisition of respiratory viruses. Alice Wimmer (International Organisation for Migration, Geneva, Switzerland) warned that migrants and refugees need to be included in national pandemic influenza preparedness plans. To comply with international human rights law, states should provide essential health services, especially disease prevention services, to migrants as well as their own nationals.

## Conclusions

Closing remarks by Julia Fitzner (WHO, Geneva, Switzerland) highlighted the need to better define severity at the individual and the population level. Predictive laboratory and digital biomarkers are needed to forecast outcomes, and data standardisation and improved interoperability of methodologies and terminologies will be required for cross-study comparisons. Methods should also be better validated.

Collaboration between providers, clinical research networks, academic groups, epidemiologists and public health agencies provides opportunities to enhance the performance of surveillance systems [10]. New data sources arising from innovative m-health tools, traditional electronic health records, participatory surveillance and web-based queries will provide additional opportunities for improvements in quality of care and (near) real-time, multi-dimensional, high-resolution surveillance.
